# Exercise training alters circadian clock dynamics in cancer‐bearing male mice

**DOI:** 10.14814/phy2.70773

**Published:** 2026-02-24

**Authors:** Loreana Sanches Silveira, Edson Alves de Lima Junior, José Cesar Rosa Neto, Alexandre Abilio de Souza Teixeira

**Affiliations:** ^1^ Immunometabolism Research Group, Institute of Biomedical Sciences University of Sao Paulo (USP) Sao Paulo Brazil; ^2^ Department of Integrative Biology & Pharmacology The University of Texas Health Science Center at Houston Houston Texas USA

**Keywords:** circadian rhythms, clock genes, inflammation, physical exercise, tumor

## Abstract

Alterations in circadian timing mechanisms are increasingly recognized as contributing to tumor initiation and progression. Moreover, evidence indicates that malignant cells can interfere with the expression and synchronization of core clock genes. Physical exercise is a potent circadian modulator in peripheral tissues, yet its effects on tumor rhythmicity remain unclear. To investigate whether the timing of exercise modulates circadian gene expression and tumor growth in a mouse model of lung cancer. Male C57BL/6J mice bearing Lewis lung carcinoma (LLC) were subjected to treadmill moderate intensity continuous training (55%–65% of maximum speed) at either a fixed (ZT2) or alternating Zeitgeber times (ZTAlt) for 3 weeks. Tumor growth, gene expression of Per1, *Per2*, *Per3*, and *Rev‐Erbα*, and TNF‐α concentrations were analyzed at six circadian time points. Rhythmic parameters were estimated using Cosinor analysis. Scheduled exercise at ZT2 significantly increased the amplitude of *Per2*, *Per3*, and *Rev‐Erbα* expression rhythms in tumor tissue. No rhythmic enhancement was observed in the ZTAlt group. TNF‐α acrophase was shifted in the ZT2 group, indicating a temporal immunomodulatory effect. Consistently performing exercise at the same time of day enhances tumor circadian clock genes rhythmicity, supporting chrono‐exercise as a potential non‐pharmacological adjuvant in cancer treatment.

## 1 INTRODUCTION

The circadian clock is an evolutionary molecular mechanism that regulates physiological processes to maintain homeostasis. This internal clock drives daily behavioral and physiological rhythms, which can be influenced by environmental factors. The modern lifestyle, characterized by an increased exposure to noise and artificial light cues, weakens the endogenous circadian rhythms' robustness (Kettner et al., 2014; Sulli et al., 2019). In humans, circadian disruption found in shift workers exposes them to an increased risk of breast (Schernhammer et al., 2001) and prostate cancer (Gan et al., 2018), although its molecular mechanisms are not fully elucidated (Masri et al., 2015). Additionally, mice with an ablation of the central clock located within the suprachiasmatic nucleus (SCN) exhibit increased tumor xenograft growth compared to mice with an intact circadian rhythm (Filipski et al., 2002).

This circadian rhythm disruption plays a fundamental role in tumor development and growth. On the other hand, oncogenic processes directly weaken the circadian rhythm (Sulli et al., 2019). For instance, the expression of all three *Per* genes is deregulated in breast cancer cells (Chen et al., 2005). *Per1* expression is downregulated in most patients, possibly due to promoter methylation.

Notably, several studies using animal models have shown an association between certain clock genes and tumorigenesis (Chen et al., 2005; Gery et al., 2005, 2006; Kelleher et al., 2014; Sahar & Sassone‐Corsi, 2007). Specifically, *Per1* and *Per2* appear to function as tumor suppressors in mice (Gery et al., 2006). The absence of *Per2* leads to the development of malignant lymphomas (Gery et al., 2005), while its expression in cancer cell lines results in cell growth inhibition, cell cycle arrest, apoptosis, and loss of clonogenic capacity (Gery et al., 2005). Interestingly, *Per2* mRNA levels are downregulated in several human lymphoma cell lines and tumor cells from patients with acute myeloid leukemia (Gery et al., 2005). Furthermore, overexpression of *Per1* can also suppress the growth of human cancer cell lines (Gery et al., 2006). Nonetheless, Per1 mRNA levels are downregulated in lung cancer compared to corresponding normal tissues (Gery et al., 2006). These findings directly point to a link between circadian rhythm disorders and cancer (Kelleher et al., 2014; Sahar & Sassone‐Corsi, 2007).

In addition to the Per family, several studies have demonstrated correlations between *Rev‐Erbα* expression and cancer (Sulli et al., 2018; Wang et al., 2018). For instance, reduced expression of *Rev‐Erbα* was observed in human gastric cancer tissues, with patient survival times significantly correlated with *Rev‐Erbα* levels. It suggests that *Rev‐Erbα* could serve as a potential prognostic factor in gastric cancer (Wang et al., 2018).

Although tumorigenesis is influenced by genetic determinants, lifestyle factors appear to be the main contributors. Behavioral risk factors, such as physical inactivity, sedentarism (Friedenreich et al., 2021), and nutritional factors are responsible for approximately one‐third of cancer deaths (Kushi et al., 2012). Physical exercise has been proposed as a strong external cue (Zeitgeber) capable of entraining circadian rhythms, particularly in skeletal muscle (Gabriel & Zierath, 2019) exercise may represent a non‐pharmacological intervention with potential to modulate cancer risk and progression (Hojman et al., 2018). However, little is known about the relationship between the mechanisms involved in physical exercise and their impact on cancer by circadian clock modulation.

Voluntary physical exercise can influence the speed at which an animal entrains to the phase of a new light/dark cycle (Edgar & Dement, 1991; Marchant & Mistlberger, 1996), thereby promoting changes in the circadian clock in skeletal muscle (Wolff & Esser, 2012). Aerobic training has been shown to enhance metabolism and performance through circadian clock mechanisms, which are disrupted in *Clock* mutant mice (Pastore & Hood, 2013).

Furthermore, there is a close relationship between genes that control circadian rhythm and various other regulatory factors important for oncogenesis, suggesting that several cancer hallmarks may be under circadian clock control (Sulli et al., 2019). Accumulating evidence indicates that regular physical activity can influence several hallmarks of cancer, including reducing tumor burden and slowing disease progression (Ruiz‐Casado et al., 2017).

Therefore, our objective was to assess the effect of physical exercise performed at consistently or at randomized times, and to examine its role as a chronomarker in the expression of clock genes in tumor‐bearing mice.

## 2 METHODS

Male C57BL/6J mice were housed under controlled conditions with a 12‐h light–dark cycle (light phase: zeitgeber (ZT) 0–12, dark phase: ZT 12–24) and a temperature maintained at 23°C ± 2°C. They were fed a standard diet (Nuvital from Nuvilab CR‐1, Colombo, PR #100110067) and had unrestricted access to water. Treatment began when the mice were between 8 and 10 weeks old. All experimental procedures were conducted under ethical guidelines for animal research and approved by the Ethics Committee on Animal Experimentation at the University of São Paulo on 08/25/2015, under registration number 95 in book 3, page 36.

The mice were categorized into three groups: Tumor control (CTRL), tumor + Exercise (Ex), with the exercise groups further divided into those training at Zeitgeber (ZT) 2 and those training at alternating times (ZTAlt). Mice in CTRL and Ex groups received subcutaneous inoculation of Lewis lung carcinoma cells. The alternating time training regimen included sessions at ZT2, ZT14, ZT6, ZT2, and ZT6. ZT0 was defined as 6:00 a.m., marking the onset of the light cycle in our facility, as presented in de Souza Teixeira et al. (2023).

Three days post‐tumor inoculation, animals underwent aerobic training via forced treadmill running. As presented in de Souza Teixeira et al. (2023), the exercise regimen was distributed over a 3‐week period, comprising a total of 14 sessions. Animals performed five sessions during each of the first 2 weeks, followed by four sessions in the final week. Each session lasted 40–60 min at 60% of maximum speed, with two rest days scheduled between weeks.

To determine maximum speed, an incremental test was conducted. Animals began with a 5‐min warm‐up at 5 m per minute (m/min), followed by an increase of 3 m/min per minute until exhaustion criteria, evidenced by observable gait changes. Specific gait alterations included repeated stumbling, uncoordinated limb movement, inability to remain upright on the treadmill belt, or gripping to the rear safety barrier for more than 3 s despite gentle prodding. Additionally, mechanical signs of fatigue such as pronounced lateral sway, irregular stride rhythm, and loss of consistent foot placement were considered indicative of exhaustion. This exhaustive test served to establish the baseline treadmill workload for subsequent training sessions and ensured that all animals were assessed under consistent, reproducible criteria (Ferreira et al., 2007).

**FIGURE 1:**
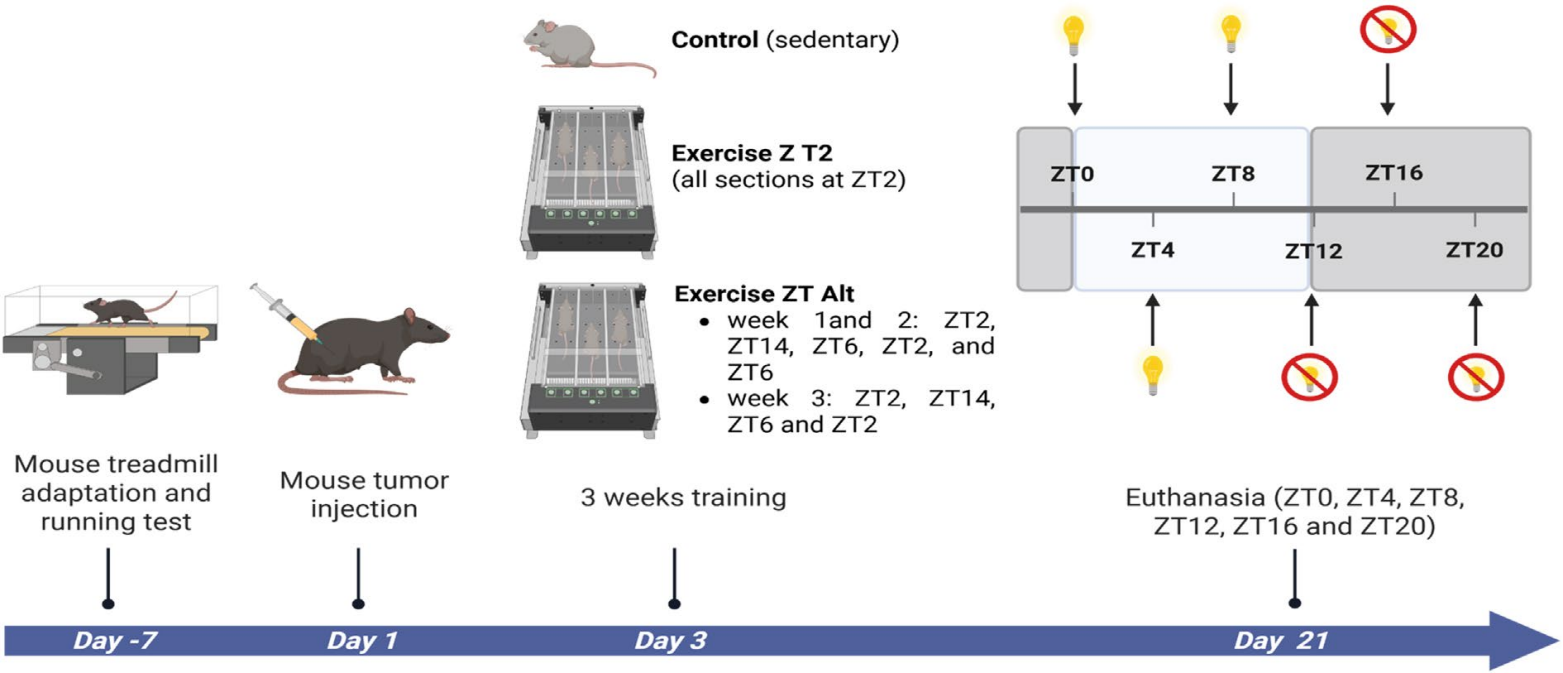
Experimental design. Made with *Biorender®*.

To explore potential circadian variations induced by tumor and/or exercise, animals were euthanized at 4‐h intervals over a 24‐h period: ZT0, ZT4, ZT8, ZT12, ZT16, and ZT20, where ZT0 marks the start of the light cycle and ZT12 the beginning of the dark cycle (Figure 1). No fasting period preceded euthanasia.

### 2.1 Subcutaneous injection of tumor cells

Lewis lung carcinoma cells, derived from mice (Kellar et al., 2015), were cultured in Dulbecco's modified Eagle's medium (DMEM, GIBCO, Invitrogen, NY), supplemented with 100 U/mL penicillin, 100 μg/mL streptomycin, and 10% fetal bovine serum (FBS, Atlanta Biologicals, Lawrenceville, GA). The cells were maintained in culture flasks at 37°C in a humidified atmosphere with 5% CO_2_. Subsequently, 5 × 10^5^ viable cells were injected subcutaneously into the right flank of mice, diluted in 0.9% sterile saline solution. Cell viability was evaluated using Trypan Blue staining.

### 2.2 RNA isolation, reverse transcription, and real‐time PCR

Total RNA was isolated from tumor tissue using Trizol reagent (TRIzol® Reagent, Life Technologies #15596026) according to the manufacturer's instructions. The extracted RNA was utilized for RT‐PCR analyses. Reverse transcription to complementary DNA (cDNA) was performed using the High‐Capacity cDNA Reverse Transcription kit (Applied Biosystems—Thermo Fisher Scientific, Foster, CA #4368813). The resulting cDNA was analyzed for *Per1*, *Per2*, *Per3*, and *Rev‐Erbα* (included in the main document), as well as *Clock*, *Bmal1*, *Cry1*, *Cry2*, and *Rorα* (presented in the Appendix S1). Real‐time PCR was conducted using Power SYBR Green PCR Master Mix (Applied Biosystems #4367659), and primer sequences are detailed in Table S1. Gene expression was quantified using the comparative method, with GAPDH used as the reference (housekeeping) gene (Livak & Schmittgen, 2001).

### 2.3 Enzyme‐linked immunosorbent assay (ELISA)

Samples of tumor tissue weighing 80–100 mg were homogenized thoroughly in RIPA buffer (0.625% Nonidet P‐40, 0.625% sodium deoxycholate, 6.25 mM sodium phosphate, and 1 mM EDTA at pH 7.4), supplemented with 10 μg/mL of protease inhibitor cocktail (Sigma‐Aldrich®, St. Louis, Missouri, USA #P8340). The resulting supernatant was utilized to determine the total protein concentration using the Bradford assay (Bio‐Rad®, Hercules, CA, USA #5000002). The concentrations of TNF‐α #DY410‐05 (reported in the main text) and IL‐6 # DY406, IL‐1β #DY401, IL‐10 #DY417 and IL‐4 # DY404 (presented in Figures S1–S4) were quantified using ELISA kits (DuoSet ELISA®, R&D Systems, Minneapolis, MN, USA) according to the manufacturer's instructions.

### 2.4 Rhythmicity analysis and statistical methods

The acrophase and amplitude were estimated for rhythmic genes using Cosinor (Cornelissen, 2014) within Discorhythm R package (R version 4.3.2 Copyright (C) 2023 The R Foundation for Statistical Computing). Data were compared using one‐way ANOVA, followed by Tukey post‐hoc analysis. Data from different groups and time points were analyzed using Two‐Way ANOVA, followed by Tukey post‐test for multiple comparisons. All experimental data are shown as mean ± Standard Deviation (SD). Statistical analyses were performed using GraphPad Prism 9.0, with significance set at *p* < 0.05. Data are presented as mean ± SD.

### 2.5 Survival analysis

The survival data of *PER2* and *PER3* genes for patients with lung cancer were obtained from Kaplan–Meier survival plots available on the Kaplan–Meier Plotter website (https://kmplot.com, accessed on July 12, 2024) (Győrffy, 2024).

## 3 RESULTS

The animals were homogeneous for total weight at the beginning of the protocol. After the protocol, following 14 sessions of physical training and 21 days post‐tumor inoculation, the Exercise ZT2 and ZTAlt groups. We also evaluated the difference between groups in terms of final body weight minus tumor weight, and a significant difference was observed between the CTRL group and both the Ex ZT2 and Ex ZTAlt groups (Table 1) (de Souza Teixeira et al., 2023). Tumor weight was significantly reduced in the Ex ZTAlt group compared with the CTRL group, corresponding to a 23% decrease in tumor burden. A more modest reduction was also observed in the exercised ZT2 group—8.25% decrease (Table 1). Adipose tissue index (the sum of inguinal, retroperitoneal and epididymal fat pads) did not present any fluctuation within the groups or when compared to pre‐ and post‐exercise protocol (Table 1).

**TABLE 1:** Net body weight (total BW−tumor weight), tumor weight, and adiposity index (Sum of retroperitoneal and epididymal adipose tissue) in grams (g) of mice after 21 days of tumor and 14 sessions of physical training.

	CRTL	EX ZT2	EX ZTAlt
Net BW (g)	24.33 ± 1.508	23.30 ± 1.670*	23.20 ± 1.730**
Tumor (g)	0.8638 ± 0.309	0.7926 ± 0.229	0.6652 ± 0.232*
Adiposity index (g)	0.333 ± 0.057	0.321 ± 0.076	0.344 ± 0.067

*Note*: The data are presented as the mean ± SD (*n* = 28–30 animals per group) *p < 0.05 and **p < 0.001 versus *CRTL* (ANOVA One‐way followed by Tukey).

**FIGURE 2:**
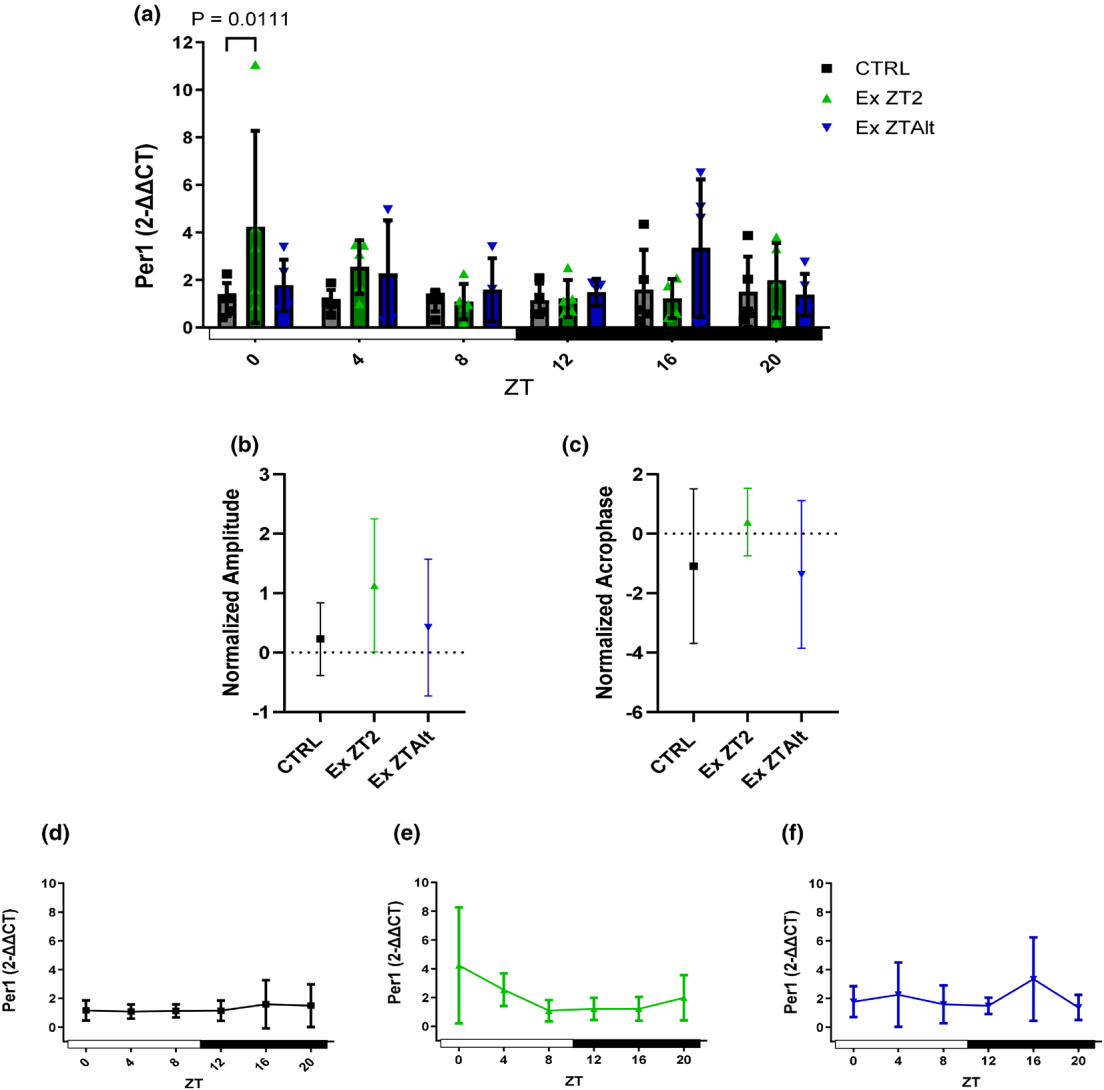
*Per1* gene expression and circadian rhythm parameters in tumor tissue of LLC‐bearing mice submitted to exercise training at the same and different times of day. (a) Relative *Per1* mRNA expression levels at six Zeitgeber times (ZT0, ZT4, ZT8, ZT12, ZT16, and ZT20) in different groups: Black = CTRL, green = Ex ZT2, and blue = Ex ZTAlt. (b) Amplitude and (c) acrophase values of *Per1* expression rhythm, estimated using the Cosinor method. (d–f) Line graphs depicting the 24‐h temporal pattern of *Per1* expression in each group individually: (d) CTRL, (e) Ex ZT2, and (f) Ex ZTAlt. Data are presented as mean ± SD from 4 to 6 animals per group. Statistical analysis: Two‐way ANOVA followed by Tukey's post hoc test for panel (a), and one‐way ANOVA followed by Tukey's post hoc test for panels (b) and (c).

**FIGURE 3:**
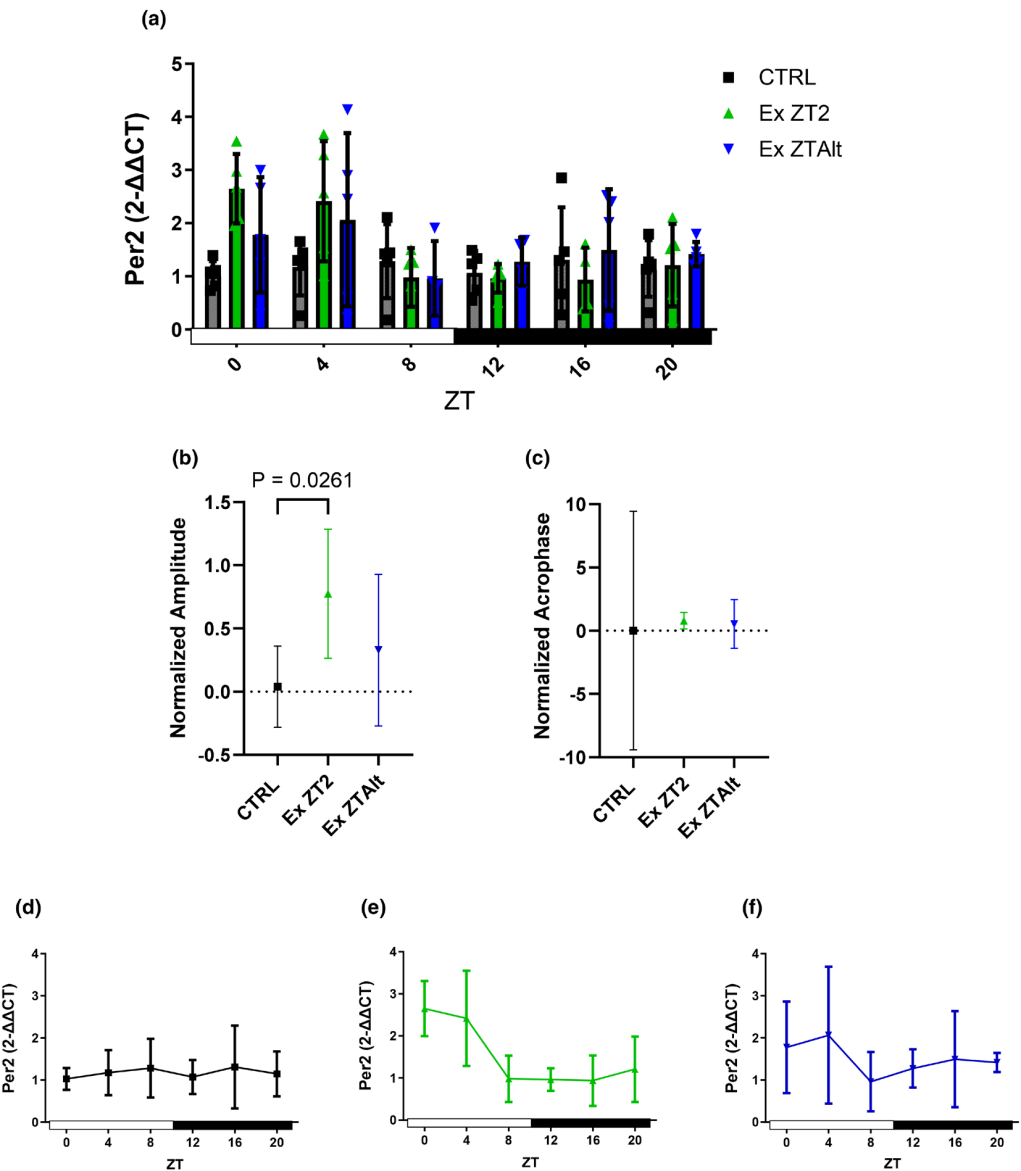
*Per2* gene expression and circadian rhythm parameters in tumor tissue of LLC‐bearing mice submitted to exercise training at the same and different times of day. (a) Relative *Per2* mRNA expression levels at six Zeitgeber times (ZT0, ZT4, ZT8, ZT12, ZT16, ZT20) in different groups: Black = CTRL, green = Ex ZT2, and blue = Ex ZTAlt. (b) Amplitude and (c) acrophase values of *Per2* expression rhythm, estimated using the Cosinor method. (d–f) Line graphs depicting the 24‐h temporal pattern of *Per2* expression in each group individually: (d) CTRL, (e) Ex ZT2, and (f) Ex ZTAlt. Data are presented as mean ± SD from 4 to 6 animals per group. Statistical analysis: Two‐way ANOVA followed by Tukey's post hoc test for panel (a), and one‐way ANOVA followed by Tukey's post hoc test for panels (b) and (c).

**FIGURE 4:**
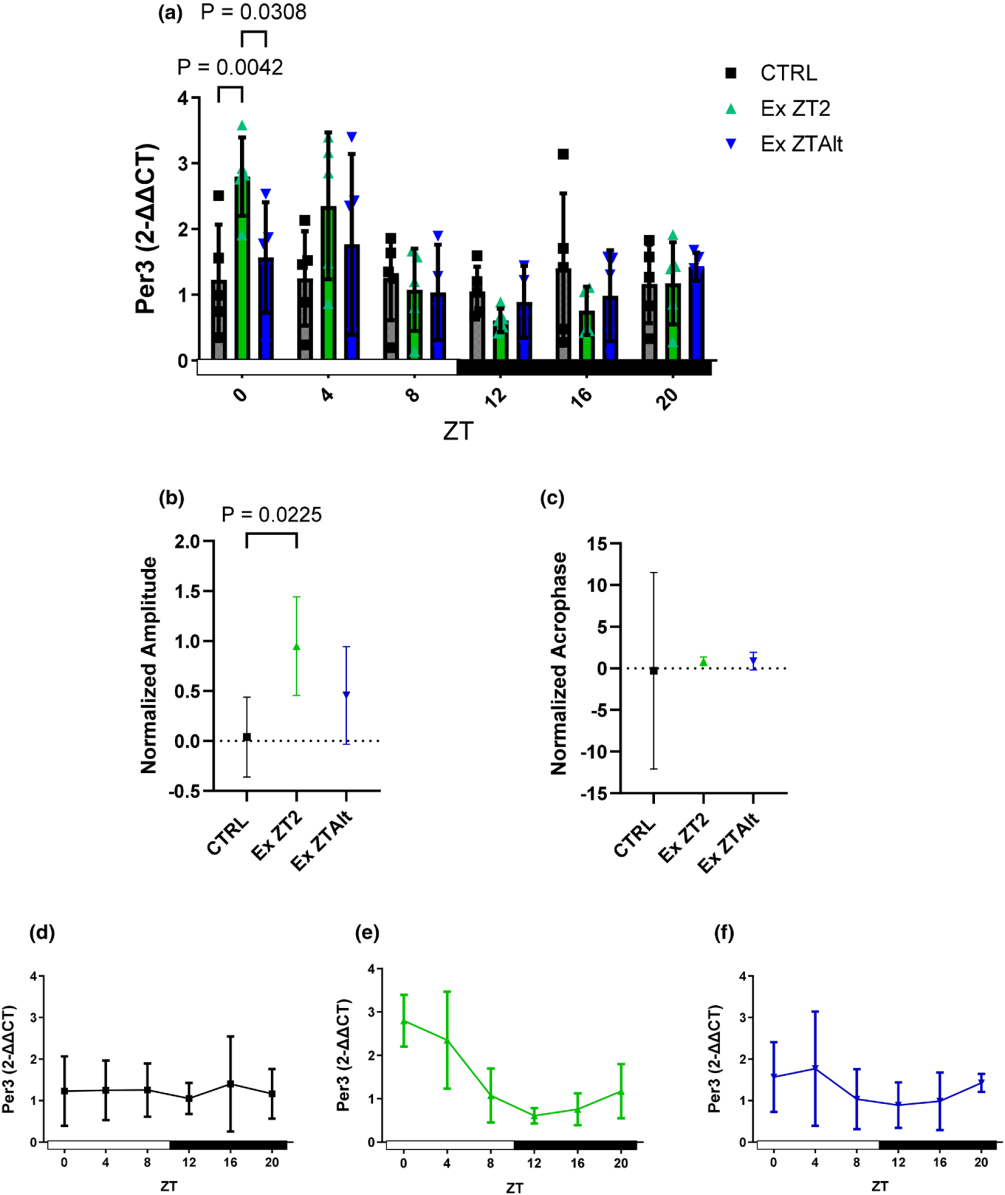
*Per3* gene expression and circadian rhythm parameters in tumor tissue of LLC‐bearing mice submitted to exercise training at the same and different times of day. (a) Relative *Per3* mRNA expression levels at six Zeitgeber times (ZT0, ZT4, ZT8, ZT12, ZT16, and ZT20) in different groups: Black = CTRL, green = Ex ZT2, and blue = Ex ZTAlt. (b) Amplitude and (c) acrophase values of *Per3* expression rhythm, estimated using the Cosinor method. (d–f) Line graphs depicting the 24‐h temporal pattern of *Per3* expression in each group individually: (d) CTRL, (e) Ex ZT2, and (f) Ex ZTAlt. Data are presented as mean ± SD from 4 to 6 animals per group. Statistical analysis: Two‐way ANOVA followed by Tukey's post hoc test for panel (a), and one‐way ANOVA followed by Tukey's post hoc test for panels (b) and (c).

Tumor gene *Per1* was higher expressed in EX ZT2 group compared to the CTRL group when assessed at ZT0. Analyses of amplitude and acrophase did not reveal any significant differences among the groups (Figure 2a–c). *Per2* showed significantly higher expression in the EX ZT2 group compared to the CTRL group at the ZT0 and ZT4 time points (Figure 3a). Additionally, the analyses of amplitude revealed significant differences between the EX ZT2 and CTRL groups (Figure 3b). *Per3* also exhibited significantly higher expression in the EX ZT2 group compared to the EX ZTAlt and CTRL groups at the ZT0 time point (Figure 4a). However, analyses of amplitude showed significant differences between the EX ZT2 and CTRL groups, with the EX ZT2 group displaying greater amplitude (Figure 4b).

**FIGURE 5:**
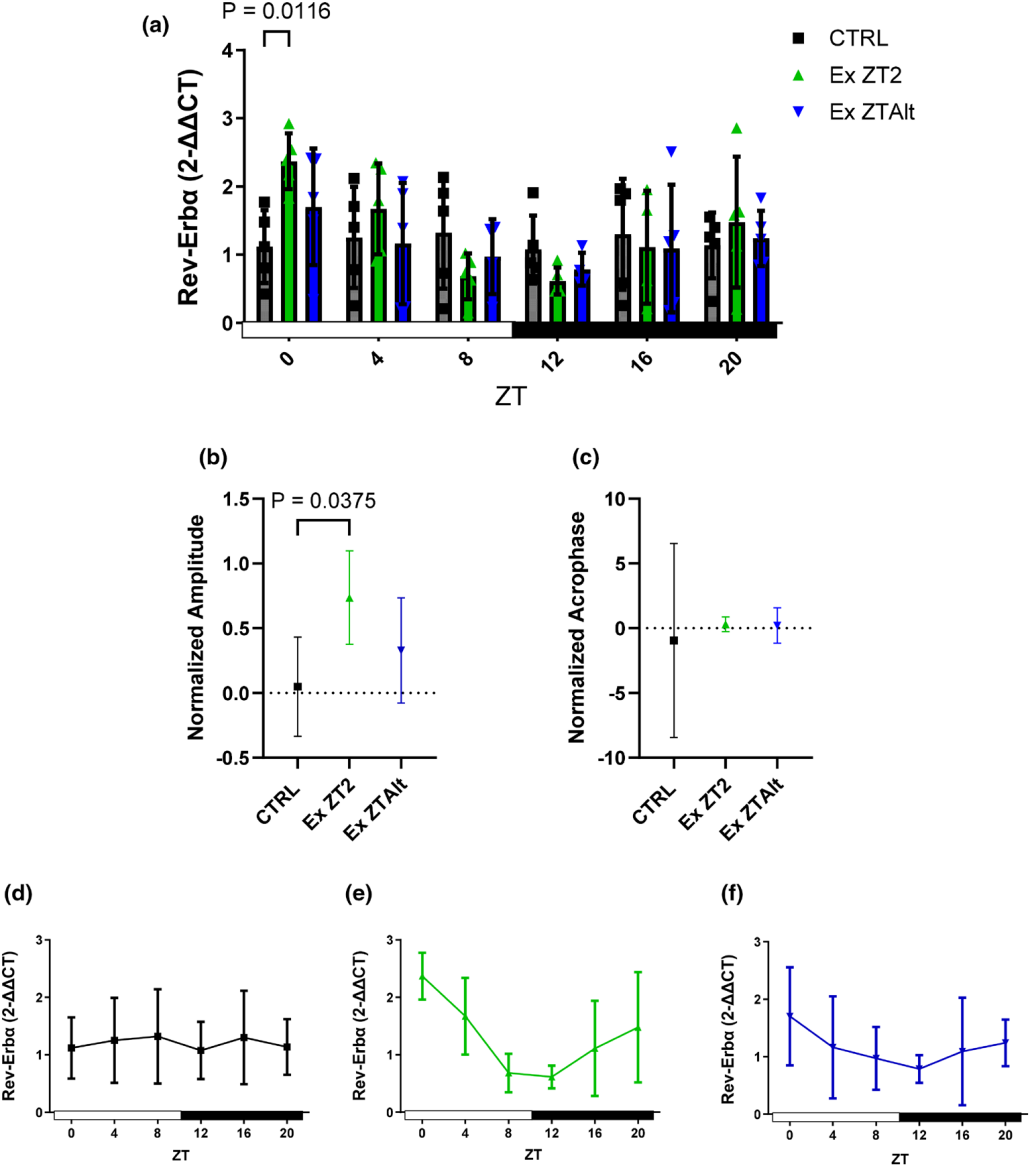
*Rev‐Erbα* gene expression and circadian rhythm parameters in tumor tissue of LLC‐bearing mice submitted to exercise training at the same and different times of day. (a) Relative *Rev‐Erbα* mRNA expression levels at six Zeitgeber times (ZT0, ZT4, ZT8, ZT12, ZT16, and ZT20) in different groups: Black = CTRL, green = Ex ZT2, and blue = Ex ZTAlt. (b) Amplitude and (c) acrophase values of *Rev‐Erbα* expression rhythm, estimated using the Cosinor method. (d–f) Line graphs depicting the 24‐h temporal pattern of *Rev‐Erbα* expression in each group individually: (d) CTRL, (e) Ex ZT2, and (f) Ex ZTAlt. Data are presented as mean ± SD from 4 to 6 animals per group. Statistical analysis: Two‐way ANOVA followed by Tukey's post hoc test for panel (a), and one‐way ANOVA followed by Tukey's post hoc test for panels (b) and (c).

*Rev‐Erbα* demonstrated significantly higher expression in the EX ZT2 group compared to the CTRL group at the ZT0 time point (Figure 5a). Consistent with these findings, analyses of amplitude showed significant differences between the EX ZT2 and CTRL groups, with the EX ZT2 group displaying greater amplitude (Figure 5b). Analyses of acrophase did not reveal any significant differences to *Per2*, *Per3*, and *Rev‐Erbα*.

A broader panel of core circadian genes, including *Clock*, *Bmal1*, *Cry*, and *Rorα*, was assessed; however, no significant alterations were detected in their rhythmic parameters, including periodicity, amplitude, or acrophase (data not shown).

**FIGURE 6:**
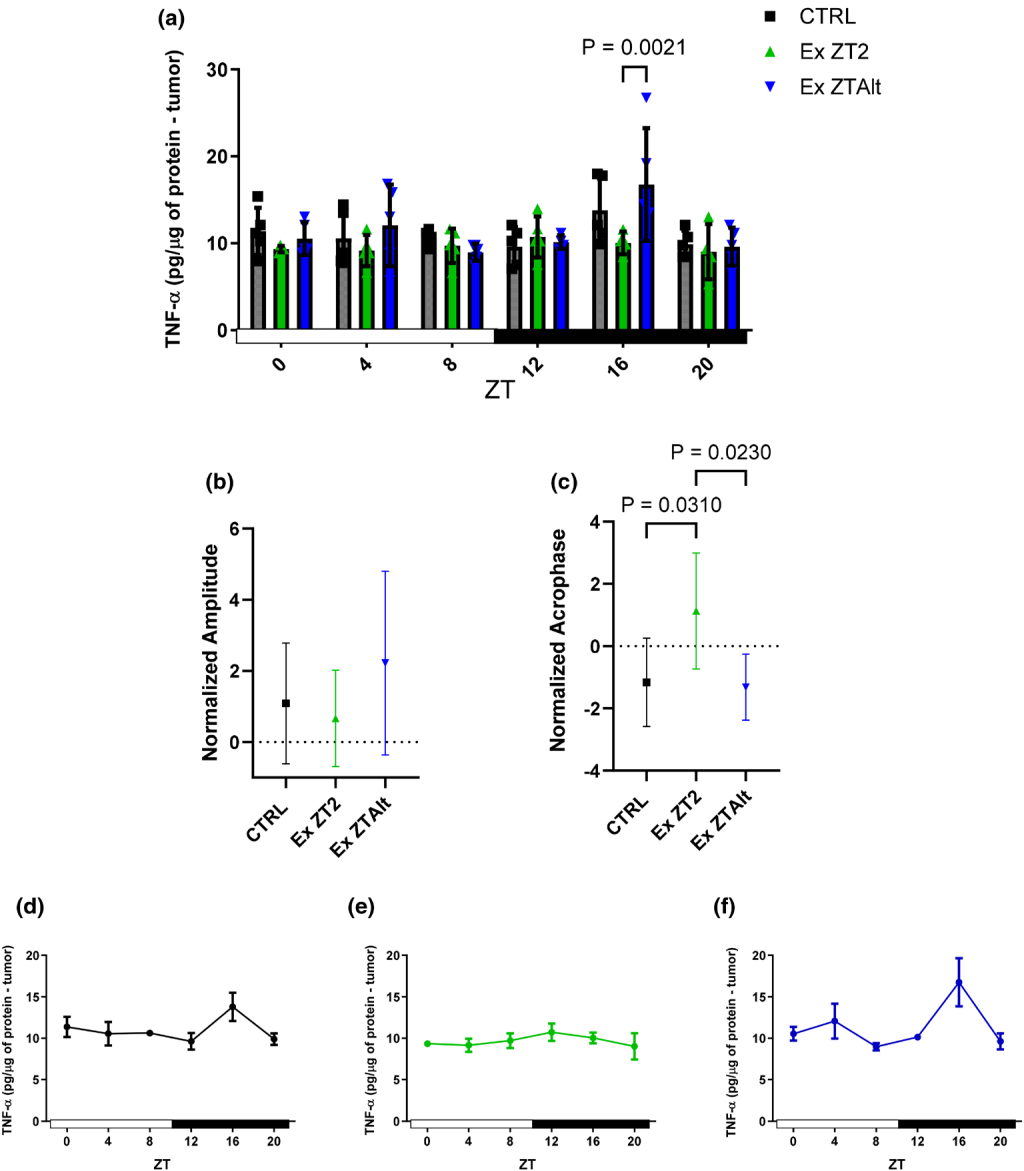
Protein concentration of TNF‐α and circadian rhythm parameters in tumor tissue of LLC‐bearing mice submitted to exercise training at the same and different times of day. (a) Relative Protein concentration of TNF‐α at six Zeitgeber times (ZT0, ZT4, ZT8, ZT12, ZT16, and ZT20) in different groups: Black = CTRL, green = Ex ZT2, and blue = Ex ZTAlt. (b) Amplitude and (c) acrophase values of protein concentration of TNF‐α, estimated using the Cosinor method. (d–f) Line graphs depicting the 24‐h temporal pattern of TNF‐α concentration in each group individually: (d) CTRL, (e) Ex ZT2, and (f) Ex ZTAlt. Data are presented as mean ± SD from 4 to 6 animals per group. Statistical analysis: Two‐way ANOVA followed by Tukey's post hoc test for panel (a), and one‐way ANOVA followed by Tukey's post hoc test for panels (b) and (c).

Regarding cytokine expression, levels of TNF‐α, IL‐6, IL‐1β, IL‐10, and IL‐4 were analyzed. While no significant differences in concentration or amplitude were detected for any cytokine (Figure S2), acrophase analysis revealed a distinct rhythm shift specifically for TNF‐α. The EX ZT2 group exhibited a significantly later acrophase compared to both the CTRL and EX ZTAlt groups (Figure 6b).

**FIGURE 7:**
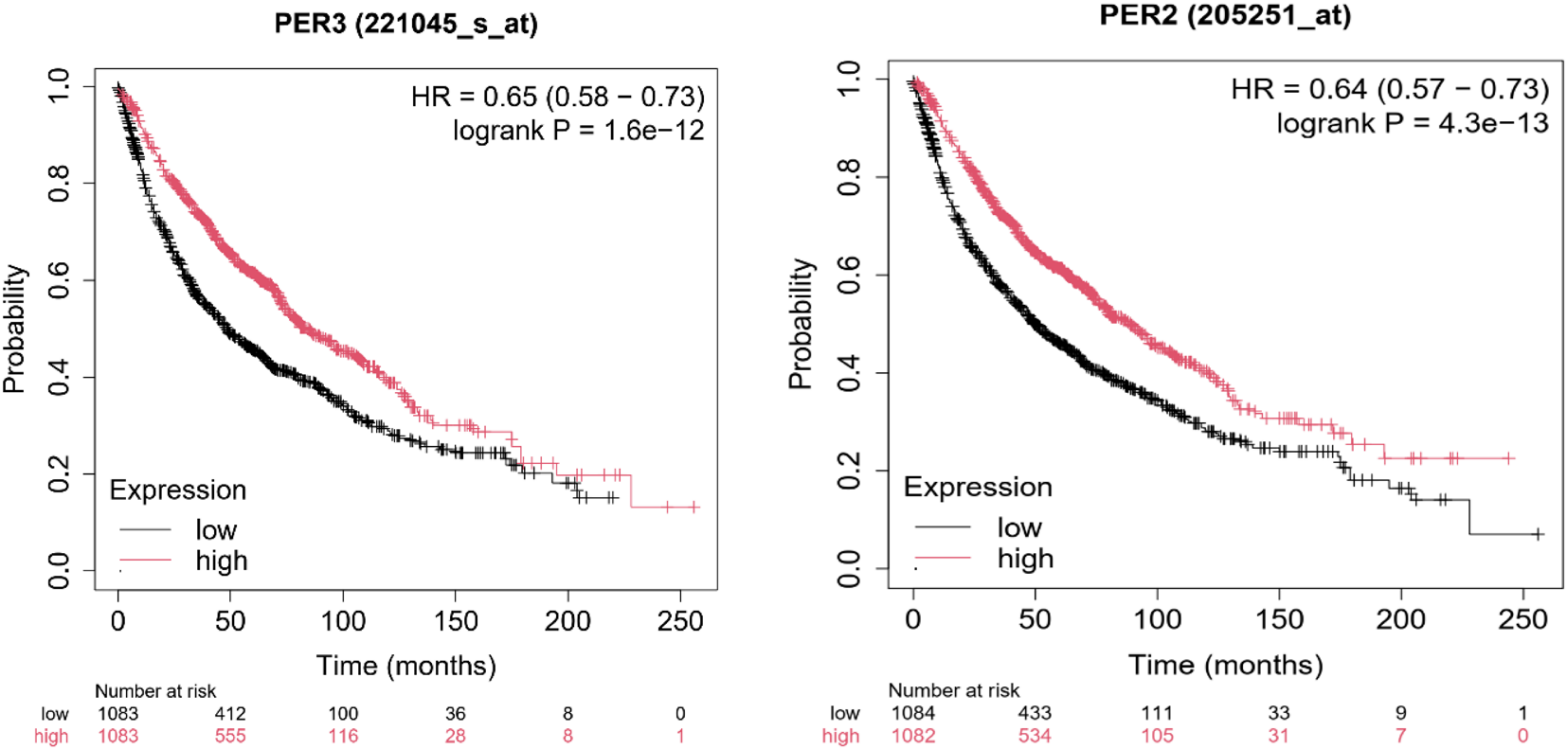
*PER2* and *PER3* genes have been linked to survival outcomes in lung adenocarcinoma. Lower expression of Per 2 and Per 3 genes is associated with shorter survival (*p* < 0.05). In graphical representation, low expression is depicted in black and high expression in red.

Finally, the genes PER2 and PER3 were evaluated using Kaplan–Meier survival analysis in human lung cancer patients to assess their clinical relevance. Higher expression levels of both genes were associated with reduced overall survival (Figure 7).

## 4 DISCUSSION

Our findings demonstrate that physical exercise performed at the same time each day was able to induce a circadian response in the *Per2*, *Per3*, and *Rev‐Erbα* genes within the tumor, significantly enhancing the amplitude of their expression.

These changes were especially evident in the groups that trained consistently at the same time each day (ZT2). In contrast, no significant alterations in gene amplitude were observed in either the sedentary group or the group that trained at randomized times. The exercise‐induced increase in *Per2* and *Per3* in LLC‐bearing mice is clinically relevant since reduced expression of the *Per* gene family has been linked to decreased overall survival in lung cancer patients (as shown in Figure 7). This underscores the potential of exercise as a modulating factor in circadian regulation within tumors, with significant implications for cancer prognosis.

Furthermore, the ZTAlt group showed changes in TNF‐α concentrations within the tumor, marked by an increased acrophase. An increase in acrophase indicates a temporal delay in the peak expression of the gene. This may reflect an adaptive adjustment that mitigates inflammation during critical periods, aligning with the well‐documented chronic anti‐inflammatory effects of exercise (Gleeson et al., 2011). In the context of cancer, this finding also suggests a promising avenue for the development of time‐based therapeutic strategies, such as chronotherapy (Li et al., 2024).

Disruption of circadian rhythm genes can influence key pathways in cancer development and progression, including cell cycle control, metabolic regulation, response to DNA damage, and apoptosis (Shafi & Knudsen, 2019; Sulli et al., 2019).

Papagiannakopoulos et al. (2016) demonstrated that disturbances in circadian rhythms may facilitate the development of lung tumors, highlighting the crucial role of circadian homeostasis as a tumor suppressor. These authors indicated that physiological and genetic disruptions (specifically *Bmal1* and *Per2* inhibition) can interact with Kras and *p53* to enhance lung tumorigenesis (Papagiannakopoulos et al., 2016). In this sense, melatonin administration was able to reduce the proliferation of a human breast cancer cell line by inducing *Per2*‐dependent p53 activation (Xiang et al., 2012). Furthermore, the systemic depletion of *Per2* has been shown to have varying impacts on the circadian behavior of animals (Zheng et al., 1999). This illustrates a strong relationship between the disruption of circadian rhythm and tumor development.

Physical exercise can be an important tool in the synchronization of the circadian rhythm (de Souza Teixeira et al., 2020) and play a significant role in cancer prevention and the management of cancer progression (Hojman et al., 2018). Conversely, cancer can induce alterations or disruptions in circadian rhythms (Sulli et al., 2019). Our study showed that physical exercise controls clock gene expression in tumor tissue, in particular, the genes *Per2*, *Per3*, and *Rev‐Erbα*.

The literature underscores the significant impact of physical exercise on cancer treatment, elucidating its multifaceted benefits. Engaging in regular physical activity has been shown to reduce fatigue (Cramp & Byron‐Daniel, 2012) and diminish chemotherapy‐related toxicities (Cave et al., 2018), thereby enhancing the overall quality of life for patients undergoing treatment. Furthermore, exercise is associated with improvements in cognitive function (Koevoets et al., 2022) and plays a critical role in mitigating skeletal muscle loss (Gould et al., 2013), which is particularly important during periods of treatment. Additionally, emerging evidence suggests that physical activity may attenuate tumor growth (Hojman et al., 2022), highlighting its potential as an integral component of comprehensive cancer care.

Engaging in early morning exercise appears to offer greater protective effects against breast and prostate cancer compared to exercising later in the day (Weitzer et al., 2021). In like manner, our data corroborated that physical exercise performed consistently at the same time of day (ZT2) showed a higher acrophase for TNF‐α concentrations compared to the other two groups. It is known that TNF‐α and its receptor can influence circadian rhythms; a study demonstrated that stimulation with LPS in mice led to increased TNF‐α concentrations, consequently causing a phase shift due to the rise in TNF‐α levels (Leone et al., 2012; Paladino et al., 2014).

The effects of TNF‐α on tumor cell death are well explored in the literature. The name of this cytokine derives from its ability to induce tumor cell death. TNF‐α was first isolated from serum and named tumor necrosis factor by Carswell and colleagues, who in 1975 discovered that this protein was released by macrophages after LPS stimulation and caused hemorrhagic necrosis in tumor cells (Carswell et al., 1975). TNF‐α can activate death signaling pathways in tumor cells by increasing apoptosis and pyroptosis, mediated by both classical and nonclassical mechanisms (van Loo & Bertrand, 2023). In addition, increased TNF‐α expression at the tumor site is associated with enhanced antitumoral immunity (Poccia et al., 1997). TNF‐α is also capable of inducing high cytotoxic activity in CD8+ T cells (Sang et al., 2024).

On the other hand, tumor development leads to elevated levels of several pro‐inflammatory mediators in the bloodstream, including TNF‐α, which is a known contributor to skeletal muscle loss (Powrózek et al., 2022; Webster et al., 2020). In our previous work, we observed that this increase in TNF‐α was localized within the tumor tissue rather than in the skeletal muscle, suggesting that exercise training elicits an anti‐inflammatory response in the muscle of LLC‐bearing mice (de Souza Teixeira et al., 2023). These findings align with previous research demonstrating exercise‐induced anti‐inflammatory effects in skeletal muscle (Lira et al., 2009). Moreover, exercise has been proposed as an adjuvant therapy in breast cancer, contributing to the attenuation of systemic inflammation (Schauer et al., 2021).

This study has several limitations. Although ZT2‐trained tumor‐bearing mice displayed more robust oscillations of core circadian genes, this did not translate into measurable differences in tumor burden at the endpoint. Given that the intervention was relatively short‐term, it is possible that the duration was insufficient to reveal statistically significant tumor reduction in the ZT2 group; longer exercise protocols may be required to elicit such effects in the absence of pharmacological treatment. Nonetheless, our findings remain consistent with evidence that tumor clock dynamics influence treatment responsiveness more strongly than baseline tumor growth, and with large‐scale analyses showing that circadian genes are frequently altered across cancers and interact with oncogenic and clinically actionable pathways (Ye et al., 2018; Zeng et al., 2014).

## 5 CONCLUSION

Daily time‐planned aerobic exercise modulates the circadian expression of *Per2*, *Per3*, and *Rev‐Erbα* genes in tumor tissue of LLC‐bearing mice, resulting in increased rhythmic amplitude despite not enhanced tumor burden. These findings highlight the potential of exercise timing as a chronotherapeutic strategy in oncology.

## AUTHOR CONTRIBUTIONS

Alexandre Abilio de Souza Teixeira: Conception and design of study, analysis and interpretation of data, and drafting the manuscript. Loreana Sanches Silveira: Conception and design of study, data analysis, and drafting the manuscript. Edson A. Lima: Conception and design of study and review of the manuscript. José Cesar Rosa Neto: Conception and design of study, analysis and interpretation of data and drafting the manuscript, funding acquisition. All authors have read and agreed to the published version of the manuscript.

## CONFLICT OF INTEREST STATEMENT

The authors declare that there is no conflict of interest regarding the publication of this paper.

## ETHICS STATEMENT

We certify that permission for the use of animals was granted to the research “Physical exercise as synchronizer of circadian rhythms in cancer”, registered as number 95, in pages 36, book 3, by the ETHICS COMMITTEE ON USE OF ANIMALS (CEUA‐ICB/USP) in 8/25/2015.

## Supporting information


Appendix S1.

## Data Availability

Data supporting the findings of this study are available from the corresponding author upon reasonable request.
