# Supplementary material for: Exercise training alters circadian clock dynamics in cancer‐bearing male mice
Source: Physiol Rep. 2026 Feb 24;14(4):e70773. doi: 10.14814/phy2.70773 (PMC12932309; doi:10.14814/phy2.70773)
Supplement: Supplementary file 1 — Appendix S1. [file PHY2-14-e70773-s001.docx]

Supplementary material

Supplementary table 1- Primer sequences - RT-PCR

| **Gene** | **Primer Forward** | **Primer Reverse** |
| --- | --- | --- |
| **Gapdh** | CAAGCTCATTTCCTGGTATGACA | GCCTCTCTTGCTCAGTGTCC |
| **Clock** | CAAGGCATGTCACAGTTTCAG | AGTTCTTCTTGCTGCCGATG |
| **Bmal1** | ACTGTTGTTTTAGCCAATGTCC | CTCTTTGGGCCACCTTCTC |
| **Per1** | GCATCCTCAGGTATTTGGAGAG | GGCTGAAGAGGCAGTGTAGG |
| **Per2** | TGAAGAACGCGGATATGTTT | GATGGAGGCCACTTGGTTAG |
| **Per3** | AACACGAAGACCGAAACAGAAT | CTCGGCTGGGAAATACTTTTTCA |
| **Cry1** | TAGCGGTGGAAATTGCTCT | GAGCTTCTCCCTTGCTTGAGT |
| **Cry2** | ACCGATGGAGGTTCCTACTG | TCCCCGGACTACAAACAGAC |
| **Rev-Erbα** | GTCTAGAGATGCTGTGCGTTTT | AGGCTGCTCAGTTGGTTGTT |
| **Rorα** | CGAGGTATCTCAGTCACGAAGA | ATTCCTGACGATTTGTCTCCAC |

**Supplementary Figure 1.** (A-J) Amplitude and acrophase values of Clock, Bmal1, Cry1, Cry2 and Rorα genes expression rhythm, estimated using the Cosinor method. *Statistical analysis: one-way ANOVA followed by Tukey’s post hoc test for panels.* Significance levels: *p < 0.05, **p < 0.01, and ***p < 0.001.*

**Supplementary Figure 2.** (A-H) Amplitude and acrophase values of protein concentration of IL-6, IL-1β, IL-10 and IL-4*,* estimated using the Cosinor method. Statistical analysis: one-way ANOVA followed by Tukey’s post hoc test for panels. Significance levels: *p < 0.05, **p < 0.01, and **p < 0.001.

**Supplementary Figure 3**. ( A-E) Clock, Bmal1, Cry1, Cry2, and Rorα gene expression in tumor tissue of LLC-bearing mice submitted to exercise training at the same and different times of day. Statistical analysis: two-way ANOVA followed by Tukey’s post hoc test. Significance levels: *p < 0.05, **p < 0.01, and **p < 0.001.

**Supplementary Figure 4**. (A-D) Protein concentration of IL-6, IL-1β, IL-10 and IL-4 in tumor tissue of LLC-bearing mice submitted to exercise training at the same and different times of day. Statistical analysis: two-way ANOVA followed by Tukey’s post hoc. Significance levels: *p < 0.05, **p < 0.01, and **p < 0.001.
